# Essential Role of Syntaxin-Binding Protein-1 in the Regulation of Glucagon-Like Peptide-1 Secretion

**DOI:** 10.1210/endocr/bqaa039

**Published:** 2020-03-06

**Authors:** Jhenielle R Campbell, Alexandre Martchenko, Maegan E Sweeney, Michael F Maalouf, Arianna Psichas, Fiona M Gribble, Frank Reimann, Patricia L Brubaker

**Affiliations:** 1 Departments of Physiology, University of Toronto, Toronto, ON, Canada; 2 Departments of Medicine, University of Toronto, Toronto, ON, Canada; 3 Wellcome Trust-MRC Institute of Metabolic Science – Metabolic Research Laboratories (IMS-MRL), University of Cambridge, Cambridge, UK

**Keywords:** enteroendocrine, exocytosis, GLP-1, glucagon, Munc18-1, SNARE, Stxbp1

## Abstract

Circadian secretion of the incretin, glucagon-like peptide-1 (GLP-1), correlates with expression of the core clock gene, *Bmal1*, in the intestinal L-cell. Several SNARE proteins known to be circadian in pancreatic α- and β-cells are also necessary for GLP-1 secretion. However, the role of the accessory SNARE, Syntaxin binding protein-1 (Stxbp1; also known as Munc18-1) in the L-cell is unknown. The aim of this study was to determine whether Stxbp1 is under circadian regulation in the L-cell and its role in the control of GLP-1 secretion. *Stxbp1* was highly-enriched in L-cells, and STXBP1 was expressed in a subpopulation of L-cells in mouse and human intestinal sections. *Stxbp1* transcripts and protein displayed circadian patterns in mGLUTag L-cells line, while chromatin-immunoprecipitation revealed increased interaction between BMAL1 and *Stxbp1* at the peak time-point of the circadian pattern. STXBP1 recruitment to the cytosol and plasma membrane within 30 minutes of L-cell stimulation was also observed at this time-point. Loss of Stxbp1 in vitro and in vivo led to reduced stimulated GLP-1 secretion at the peak time-point of circadian release, and impaired GLP-1 secretion ex vivo. In conclusion, Stxbp1 is a circadian regulated exocytotic protein in the intestinal L-cell that is an essential regulatory component of GLP-1 secretion.

Current frontline therapies for patients with type 2 diabetes include glucagon-like peptide-1 (GLP-1) mimetics that not only harness the insulin-stimulating and glucagonostatic properties of the endogenous hormone, but also lower body weight through induction of satiety (1). A potential new class of GLP-1-based therapies currently being investigated involves increasing the endogenous secretion of GLP-1 (2). However, full development of such an approach requires identification and further understanding of the molecular pathways regulating GLP-1 secretion by the intestinal L-cell.

L-cells are scattered throughout the intestinal epithelial layer of the gut, but are predominantly localized to the distal ileum and colon (3). GLP-1 secretion from these cells is stimulated by nutrient ingestion, as well as by a variety of endocrine hormones and adipokines, bile acids, and intestinal microbial metabolites (4, 5). Recent studies have demonstrated that GLP-1 secretion is also modulated by the circadian clock, demonstrating peak release at the onset of the dark, active period in rodents, and differential release by time of day in humans (6–10). Circadian secretion of GLP-1 has also been demonstrated in both murine and human L-cell models in vitro, and both suppression and knockout of the circadian clock gene, *Bmal1*, result in dampened GLP-1 release (6, 8, 11). However, although the actions of the various L-cell secretagogues are mediated by different signaling pathways, they must all converge on the basolateral side of the enteroendocrine L-cell to induce exocytosis of the GLP-1-containing secretory granules (12).

Regulated exocytosis involves the core SNARE proteins (syntaxin, SNAP, and VAMP), in addition to a host of accessory and regulatory proteins (13, 14). Within the intestinal L-cell, previous studies have identified the core SNAREs, VAMP2 and syntaxin1a (Stx1a), and 2 calcium-regulated accessory SNAREs, synaptotagmin-7 and secretagogin, as essential for GLP-1 secretion (10, 15–17). Furthermore, the regulatory SNARE protein, syntaxin-binding protein-1 (Stxbp1; also known as Munc18-1), coimmunoprecipitates with VAMP2, Stx1a, and SNAP25 in the murine (m) GLUTag L-cell line (15, 18).

Recent studies in pancreatic α- and β-cells have demonstrated the existence of circadian rhythms in VAMP2, SNAP25, synaptotagmin-7, and Stxbp1, in association with circadian secretion of the associated hormones, glucagon and insulin, respectively (19, 20). However, although β-cell *Stxbp1* knockdown (KD) results in impaired exocytosis of insulin (21), nothing is known about the regulation of its expression or its role in GLP-1 secretion by the intestinal L-cell. Notwithstanding, recent microarray and mass spectrometry analyses of the mGLUTag intestinal L-cell line have demonstrated that Stxbp1 expression is significantly increased at the peak time-point of circadian GLP-1 release compared with the trough time-point of secretion (10). The goal of the present study was, therefore, to determine whether Stxbp1 expression is regulated in a circadian fashion in the intestinal L-cell and to establish whether this regulatory SNARE protein is required for GLP-1 secretion.

## Materials and Methods

### In vitro studies

mGLUTag (RRID: CVCL_J406) (22) L-cells are a clonal line derived from the distal intestine of a male mouse (18), which respond to GLP-1 secretagogues and retain an autonomous circadian clock (6, 23–27). mGLUTag L-cells were grown in DMEM containing 10% fetal bovine serum (FBS). Two days after plating, they were serum-starved for 12 hours in 0.5% FBS, synchronized by a 1-hour shock with 20 µM forskolin in 10% FBS, and then incubated for up to 48 hours in 10% FBS (6, 8, 10, 11).

For STXBP1 localization analyses, mGLUTag L-cells were grown on multiwell microscope slides, synchronized as described previously, and then stimulated at the 8-hour time-point with 50 µM forskolin plus 50 µM 3-isobutyl-1-methylxanthine (IBMX; to chemically increase cAMP levels) in 10% FBS or vehicle (control), for up to 60 minutes.

Small interfering RNA (siRNA)-mediated reverse-transfection was used to KD *Stxbp1* in mGLUTag L-cells. Transfection media with 50 nM siRNA or scrambled RNA (scRNA; SMART pool: ON-TARGETplus Stxbp1 siRNA or ON-TARGETplus Non-targeting Pool, respectively; Lafayette, CO) and 0.75 µL Dharmafect 3 in 10% FBS was placed into the wells and the mGLUTag L-cells were then passaged directly into the transfection-media and incubated for 72 hours. Cells were studied immediately or were then serum-starved, synchronized and incubated for 8 hours, as described; the respective siRNA and scRNA were included in the media during the serum-starvation and 8-hour incubation steps to maintain the KD (data not shown). Cells were used for RNA and protein extraction and GLP-1 secretion assay.

For GLP-1 secretion assay, mGLUTag L-cells were synchronized and incubated for 8 hours in media with 10% FBS, as previously described, and then treated with 10^-7^ M glucose-dependent insulinotrophic polypeptide (GIP; to physiologically increase cAMP levels) or media alone (control) for 2 hours. Each treatment group comprised 8 wells derived from 2 separate splits, to make n = 8. Peptides contained in the media and cells were collected using a C18 SepPak (Waters Associates, Milford, MA), as reported (6, 24–27), and GLP-1 levels were determined by Total GLP-1 Radioimmunoassay (GLP-1T-36HK, Millipore, Etobicoke, ON, Canada). Secretion was expressed as the percentage of the media GLP-1 content over the total GLP-1 content (media + cells).

### In vivo studies

C57BL/6J *Stxbp1* floxed (*Stxbp1*^fl/fl^) mice were a generous gift from Dr. M. Verhage (Vrije Universiteit, Amsterdam, Netherlands (28)) and were crossed with C57BL/6J *Gcg-CreER*^*T2/+*^*;Rosa26-GCamP3*^*fl/fl*^ mice (*Gcg-CreER*^*T2/+*^; Fig. S1A and Supplemental Methods at the following URL: http://hdl.handle.net/1807/98201 (29)). The heterozygotes were backcrossed to *Stxbp1*^fl/fl^ mice to generate inducible *Gcg-CreER*^*T2/+*^*; Rosa26-GCamP3*^*fl/fl*^*;Stxbp1*^*fl/fl*^ knockout (KO) mice. Mice that were homozygous for the *Stxbp1* floxed gene and positive for *Gcg-CreER*^*T2/+*^ were classified as inducible L-cell *Stxbp1* KO mice (Table S1 and Fig. S1B in File Inventory (29)); expression of the GCamP3 reporter was not a consideration for the current study. KO was induced by intraperitoneal injection of tamoxifen (1 mg/d) in sunflower oil (vehicle) for 5 days. Control animals included *Gcg-CreER*^*T2/+*^*;Stxbp1*^*fl/fl*^ mice treated with vehicle, and *Stxbp1*^*fl/f*l^ and *Gcg-CreER*^*T2/+*^ animals both with and without tamoxifen (Fig. S1C in File Inventory (29)). All animals were housed under a 12:12 light:dark cycle (lights on at 0600; zeitgeber time [ZT] 0) and were fed ad libitum. The study included both male- and female-matched, litter- and/or colony-mates at 7 to 12 weeks of age (except for the study analyzing GIP levels, in which mice were 8 to 20 weeks of age). All breeding and experimental procedures were approved by the Animal Care Committee at the University of Toronto and all procedures followed Canadian Council on Animal Care guidelines.

An oral glucose tolerance test (OGTT) was performed 7 to 9 days following treatment with tamoxifen or vehicle (6, 24, 27). KO and control mice were fasted for 4 hours before the OGTT, which was conducted at the established peak GLP-1 secretory time-point in mice (ZT14) (10), in the dark under a red light. A glucose meter (OneTouch, LifeScan, Burnaby, BC, Canada) was used to measure blood glucose levels from tail vein blood, before oral gavage of 40% glucose (4 g/kg body weight) at t = 0 min, and then at t = 10, 30, 45, 60, 90, and 120 minutes (of note, pilot studies indicated that a 5 g/kg glucose load in this strain of mice resulted in glycemia that was above the limit of detection; data not shown). Tail vein blood (100 µL) was collected into EDTA at t = 0, 10, and 60 minutes for measurement of plasma total GLP-1 and insulin levels using the Meso Scale Discovery Multi-Spot Assay (Meso Scale Diagnostics, Rockville, MD (6)), and glucagon or GIP by ELISA (CrystalChem, Elk Grove Village, IL).

One-day following the OGTT, mice were weighed and sacrificed, and the small intestine and colon were weighed and the lengths taken under contact tension. Two centimeters sections were flash-frozen for molecular analysis or fixed in 10% formalin for imaging. A segment of ileum was also used to prepare primary cell cultures, as discussed next.

### Ex vivo studies

Adult mouse ileal crypt cell cultures were derived from approximately 10 cm of ileum, as previously reported (16). In brief, isolated crypts were suspended in 10% FBS-DMEM (with 2 mM L-glutamine, 100 U/mL penicillin, 0.1 mg/mL streptomycin, and 0.1% v/v ROCK Inhibitor [Y-27632, Sigma-Aldrich]) and crypts from 1 ileum were plated onto a 24-well plate coated with Corning Matrigel (ThermoFisher Scientific) and incubated at 37°C with 5% CO2.

GLP-1 secretion assays were conducted 24-hour after crypt plating, as reported (16). Briefly, the cells were washed and then treated with 50 µM forskolin/50 µM IBMX or media alone (control) for 2 hours. Four wells were tested for each treatment group to make n = 1 per mouse. GLP-1 concentrations in the media and cell lysates were measured using a GLP-1 (active) ELISA kit (Millipore). Secretion was expressed as a percentage of the media GLP-1 content over the total GLP-1 content (media + cells).

### Analyses

#### RNA-seq.

The methods for fluorescent assisted cell sorting of L- and EEC-cells, using either the expression of a yellow fluorescent reporter in transgenic GLU-Venus mice or NeuroD1-Cre x Rosa26EYFP mice (a generous gift from Andrew Leiter) or immunostaining of human tissue isolates with fluorescent antibodies, have been previously described (30). RNA isolation and sequencing are described elsewhere (30, 31) and the data are deposited in the NCBI GEO repository (human, GSE114853; mouse, GSE114913). The studies were conducted in accordance with the principles of the Declaration of Helsinki and good clinical practice. Human ethical approvals were given by Cambridge Central and South Research Ethics Committees (ref: 09/H0308/24, 16/EE/0338, 15/EE/0152) and the Inserm ethics committee and Agence de la biomédecine (ref: PFS16-004). Animal work was regulated under the Animals (Scientific Procedures) Act 1986 Amendment Regulations 2012 and conducted following ethical review by the University of Cambridge Animal Welfare and Ethical Review Body.

#### Chromatin-immunoprecipitation (ChIP).

Two canonical E-box sequences (CACGTG) were identified at 2016 and 646 bp upstream of the 5′ transcription start site of the *Stxbp1* gene. ChIP was conducted using the SimpleChIP Enzymatic Chromatin IP Kit with Magnetic Beads #9003 (Cell Signalling) according to the manufacturer’s instructions and as previously described (10). Briefly, mGLUTag L-cells were fixed at 8 and 20 hours after cell synchronization, followed by DNA digestion and incubation with Bmal1 antibody (Cell Signaling Technology). Protein was precipitated using protein G magnetic beads and DNA was eluted and quantified by RT-quantitative PCR (RT-qPCR) (Table S2 in File Inventory (29)). Signal from each immunoprecipitation was calculated as a percent of the total input chromatin using the following formula: Signal relative to input=2%×.

#### RNA isolation and gene expression analysis.

Total RNA was isolated from cells and tissue using the PARIS (Invitrogen, Burlington, ON, Canada) and Qiashredder/RNeasy Plus (Qiagen Inc., Toronto, ON, Canada) kits, respectively. RNA was then reverse-transcribed and DNAse-treated using 5X-All-In-One RT MasterMix with AccuRT Genomic DNA Removal Kit (Applied Biological Materials Inc., Richmond, BC, Canada). Gene expression was quantified using qPCR with TaqMan Fast Advanced Master Mix (Table S3 in File Inventory (29); ThermoFisher Scientific). Data were analyzed using the ΔΔCT method with Histone3a or 18S as the internal control for cells and tissues, respectively (32).

#### Protein isolation and immunoblot.

Protein from the mGLUTag L-cells was isolated using the PARIS (Invitrogen) kit and quantified by Bradford Assay. Samples were run on a 10% SDS-PAGE gel, transferred to a polyvinylidene fluoride membrane and incubated overnight at 4°C with anti-STXBP1 or anti-BMAL1 and anti-HISTONE H3 (loading control) antisera in TBST-T (33–35); molecular weights were determined with a biotinylated ladder (Table S4 in File Inventory (29)). Blots were then incubated with HRP-linked secondary antibodies for 1 hr (36, 37), and imaged using Signalfire Elite Enhanced Chemiluminescent reagent (New England Biolabs, Whitby, ON, Canada).

#### Immunocytochemistry.

After treatment for up to 60 minutes with forskolin/IBMX, the plasma membrane of live mGLUTag L-cells was labelled with 2.5 µg/μL Wheat Germ Agglutinin-Alexa Fluor 488 Conjugate (ThermoFisher Scientific) in Hanks’ balanced salt solution at 37°C for 10 minutes. Cells were then fixed in paraformaldehyde, permeabilized with Triton X-100, and blocked with BSA before applying anti-STXBP1 antibody (BD Bioscience, San Jose, CA (33)) for 2 hours at 20°C (Table S4 in File Inventory (29)). After washing, cells were incubated with AlexaFluor555-coupled secondary antibody for 1 hour (ThermoFisher Scientific (38)), mounted with Vectashield containing DAPI (Vector Laboratories, Burlington, ON, Canada), and imaged using a Nikon Swept Field Confocal microscope and NIS-Elements Imaging software (Nikon Corporation, Melville, NY). STXBP1 expression and localization were determined by: (1) the ratio of the intensity of STXBP1 expression in randomly selected, defined regions of interest in the cytoplasm and nucleus (i.e., cytoplasmic STXBP1 intensity/cytoplasmic area) / (nuclear STXBP1 intensity/nuclear area); and (2) Pearson’s correlation coefficient for colocalization of STXBP1 immunostaining with staining by the membrane marker.

#### Immunofluorescence.

Formalin-fixed paraffin-embedded normal and L-cell STXBP1 KO mouse ileal and colonic sections (UHN Pathology Services, Toronto, ON, Canada) and normal human ileal sections (OriGene, Rockville, MD; sample ID #PA11B41805, PA00009BED, PA00000353, PA00000426, PA15476907, and PA15476916) were costained for STXBP1 and GLP-1 by antigen-retrieval using Retrievagen A (BD Bioscience), blocking and then incubation with anti-STXBP1 and anti-GLP-1 antibodies overnight at 4°C (Table S4 in File Inventory (29)). AlexaFluor488- and 555-coupled secondary antibodies were then applied for 1 hour, followed by imaging as described previously (33, 38–40).

#### Statistical analyses.

Data are presented as mean ± SEM. The statistical difference between 2 groups was calculated by Student *t*-test, and by 1- or 2-way ANOVA followed by a Tukey post hoc test for data with 3 or more groups in Microsoft Excel and GraphPad Prism, respectively. The 24- to 48-hour gene expression data were analyzed using JTK_CYCLE for determination of circadian significance and period (11).

## Results

### GLP-1 and Stxbp1 are coexpressed in a sub-population of primary mouse and human intestinal L-cells

**Figure 1.**
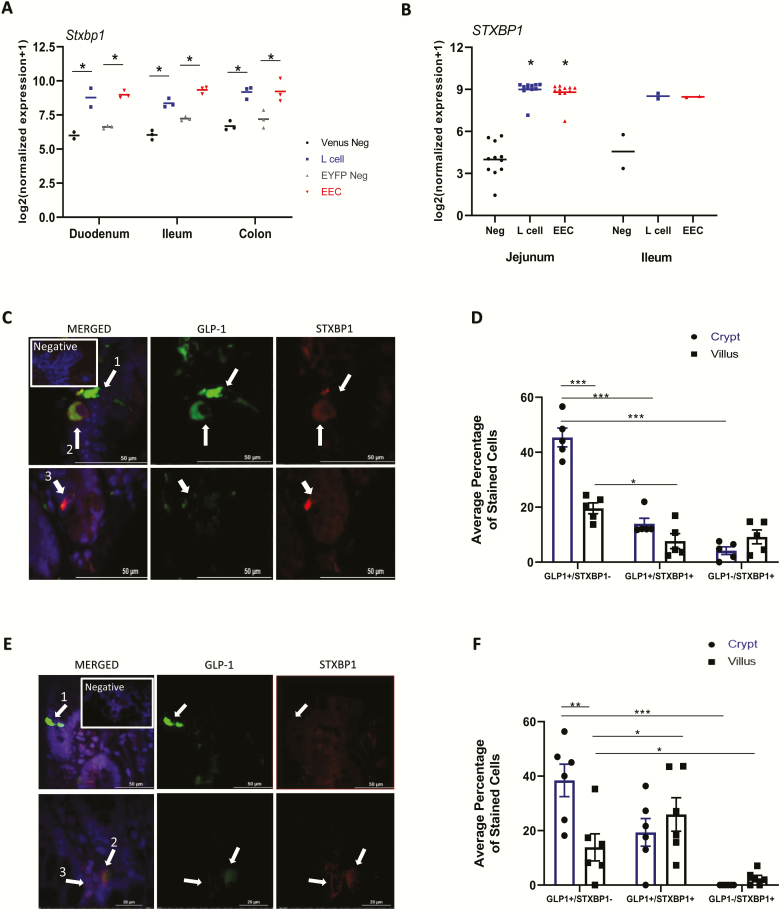
**GLP-1 and Stxbp1 are coexpressed in a subpopulation of primary mouse and human intestinal L-cells.** RNA-seq for *Stxbp1* in isolated murine Gcg-Venus L-cells, or general enteroendocrine cells (EEC), including L-cells (NeuroD1^EYFP^), and surrounding intestinal epithelial cells (A), dominated by nonenteroendocrine enterocytes (negative controls; neg), from mouse (n = 2–3) and (B) from L-cells, other enteroendocrine (excluding L-cells) and nonenteroendocrine cells from human (n = 2-11) sections along the intestine as indicated. Statistical analysis by 2-way ANOVA for murine (grouped according to tissue and fluorescent positive vs negative for GLU-Venus and NeuroD1^EYFP^ sorts separately) and 1-way-ANOVA for human tissue. **P < 0.01, ***P < 0.001. (C-F) Immunostaining of (C-D) mouse (n = 4) and (E-F) human (n = 6) ileal sections for GLP-1 and STXBP1; DAPI (blue) shows the nuclei. (C,E) Representative images and the proportion of cells staining for each marker in the crypt (blue bars) and (D,F) villi (black bars) are shown. *P < 0.05, **P < 0.01, ***P < 0.001.

RNA-seq analysis of primary L-cells from murine and human intestines demonstrated marked enrichment of L-cell *Stxbp1* and *STXBP1* transcript levels, respectively, as compared with non-L-cells throughout the small intestine and colon of mice as well as in the small intestine of humans (Fig. 1A, B). However, *Stxbp1* and *STXBP1* were not exclusive to the L-cell, demonstrating expression in other enteroendocrine cells in humans and likely in mice, although the signal observed in NeuroD1-EYFP enteroendocrine cells might have arisen partially from L-cells included in this population as, in a recent single-cell RNA-seq study of colonic endocrine cells, *Stxbp1* expression was equivalent in L- and somatostatin-expressing D- and serotonin-producing enterochromaffin-cells (41).

Costaining of mouse ileal tissue for GLP-1 and STXBP1 revealed 3 distinct populations of cells in the epithelium: cells that expressed only GLP-1 (GLP1^+^/STXBP1^-^), those that coexpressed both GLP-1 and STXBP1 (GLP1^+^/STXBP1^+^), and a third population that expressed STXBP1 only (GLP1^-^/STXBP1^+^; Fig. 1C, D). In accordance with current literature (42), the total number of GLP1^+^ cells was greater in the crypt than the villus (59.4 ± 3.9% for mouse, *P* < 0.01; 57.8 ± 7.8% for human). However, notably, many of the GLP1^+^ L-cells in the mouse ileum did not express detectable levels of immunoreactive STXBP1 (*P* < 0.001 and *P* < 0.05 for villus and crypt, respectively), whereas an even smaller percentage of the cells were STXBP1^+^ but GLP1^-^ (*P* < 0.001 for villus; Fig. 1C, D). Costaining of human ileal sections revealed the same 3 populations of cells, with similar patterns in GLP-1 and/or STXBP1 expression, except that the reduction in GLP-1^+^/STXBP1^+^ cells in the villus did not reach significance (Fig. 1E, F).

### Stxbp1 demonstrates a circadian pattern of expression in mGLUTag L-cells regulated by BMAL1

**Figure 2.**
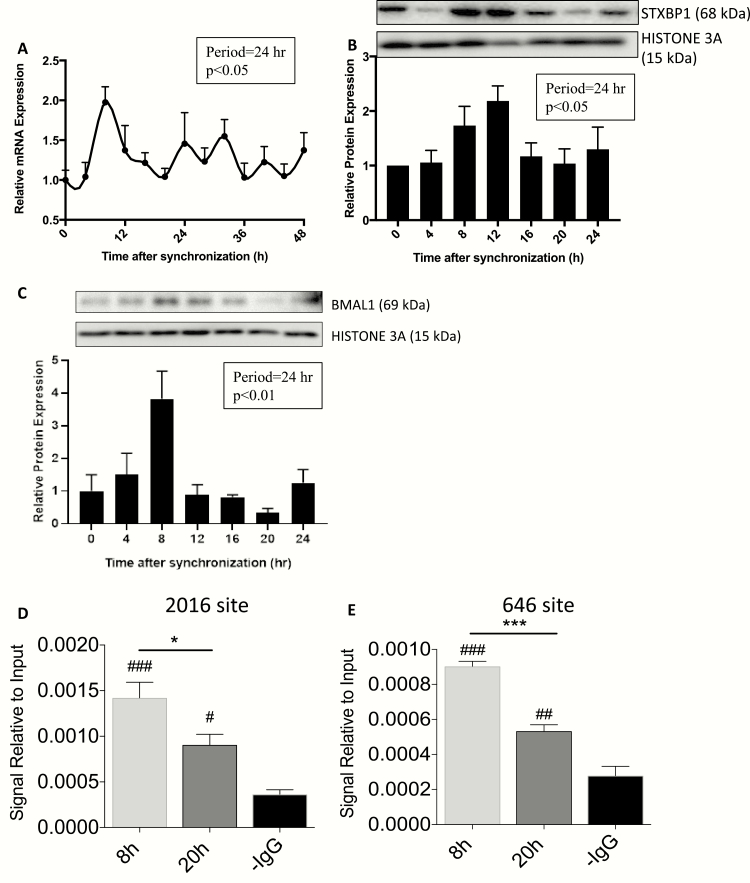
**Stxbp1 demonstrates a circadian pattern of expression in mGLUTag L-cells that is regulated by BMAL1.** mGLUTag L-cells were synchronized and analyzed for up to 48 hours by (A) RT-quantitative PCR for *Stxbp1* and by (B) Western blot for STXBP1 and (C) BMAL1. (D-E) mGLUTag L-cells were analyzed by ChIP, at 8 and 20 hours following synchronization, for BMAL1 binding to the E-boxes at (D) -2016 bp and (E) -646 bp in the *Stxbp1* promoter. ***P < 0.001; ##*P* < 0.01, ###*P* < 0.001 vs negative control (-IgG).

As we have previously reported higher expression of both *Stxbp1* transcript and STXBP1 protein levels at the peak time-point of GLP-1 secretion as compared with the trough (i.e., 4-8 vs. 16-20 hours after synchronization, respectively) (6, 10), synchronized mGLUTag L-cells were analyzed over 48 hours for the existence of a circadian rhythm. *Stxbp1* levels peaked at 8 and troughed at 20 hours, demonstrating a significant rhythm with a period of 24 hours (*P* < 0.05; Fig. 2A) that dampened over time, as well-established to occur for the circadian clock in other cells in vitro (43). Consistent with this finding, STXBP1 demonstrated a 4-hour translational delay, peaking at 12 hours with a 24-hour period (*P* < 0.05; Fig. 2B). Additionally, the *Stxbp1* rhythm was identical to that observed for BMAL1 expression, which peaked at 8 hours and troughed at 20 hours (*P* < 0.01; Fig. 2C) as previously reported (6, 10).

ChIP analysis was conducted to determine whether BMAL1 might directly regulate *Stxbp1* expression. BMAL1 binding to each of the E-boxes was detected at both 8 and 20 hours after mGLUTag L-cell synchronization (*P* < 0.001); however, binding was significantly greater at 8 hours compared to 20 hours for each E-box (*P* < 0.001; Fig. 2D, E). All further studies were therefore conducted at the peak time-point of GLP-1 secretion, 8 hours postsynchronization in mGLUTag L-cells and ZT14 (8 pm) in vivo (6, 10).

### STXBP1 is translocated to the mGLUTag L-cell cytosol and plasma membrane upon stimulation of GLP-1 secretion

**Figure 3.**
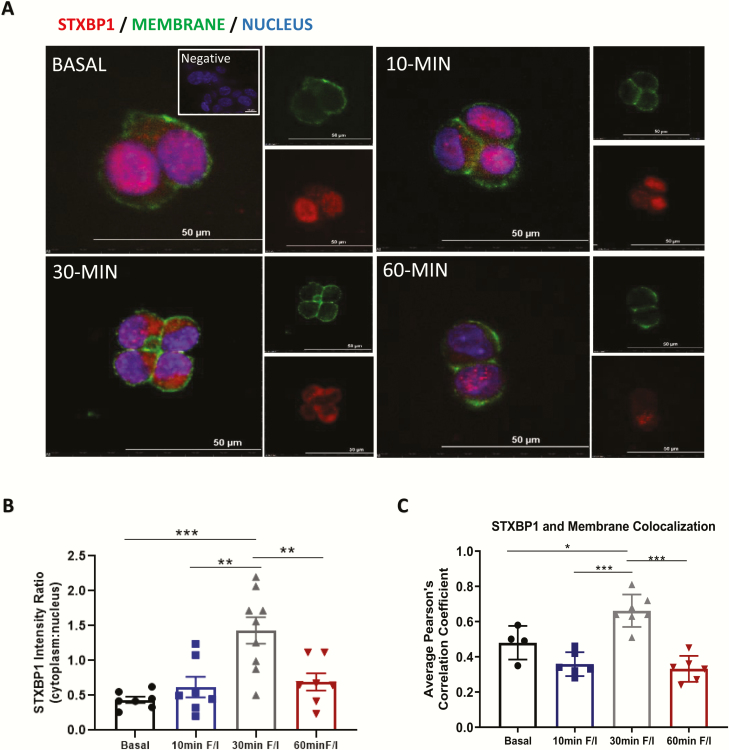
**STXBP1 is translocated to the mGLUTag L-cell membrane upon stimulation of GLP-1 secretion.** Eight hours after synchronization, mGLUTag L-cells were treated with 50 µM forskolin (F) plus 50 µM IBMX (I) for up to 60 minutes and then immunostained for STXBP1; Wheat Germ Agglutinin was used for the membrane marker. (A) Representative images, (B) STXBP1 cytoplasmic to nuclear intensity ratio (n = 7–9) and (C) Pearson correlation coefficient for colocalization of STXBP1 with the membrane marker (n = 4). *P < 0.05, **P < 0.01, ***P < 0.001.

To determine whether STXBP1 localization is altered upon stimulation of GLP-1 secretion, an acute temporal analysis was conducted in mGLUTag L-cells 8 hours after synchronization. Under basal conditions, similar levels of STXBP1 expression were observed in the cytoplasm and nucleus (Fig. 3A, B). However, this pattern changed at 30 minutes (but not at 10 or 60 minutes) after stimulation with the known secretagogue, forskolin (16, 23), with a 225% increase in immunoreactivity detected in the cytoplasm (*P* < 0.001). STXBP1 immunoreactivity was also detected at the cell membrane under basal conditions, colocalizing with the membrane marker (Pearson correlation coefficient = 0.48, Fig. 3A, C). However, after 30 minutes of stimulation, there was again a change in STXBP1 localization, with a 38% increase in expression at the plasma membrane (*P* < 0.05). These subcellular shifts in STXBP1 with stimulation suggest recruitment toward the plasma membrane in association with increased GLP-1 exocytosis.

### KD of Stxbp1 in mGLUTag L-cells impairs GLP-1 secretion in vitro

Pilot studies in unsynchronized mGLUTag L-cells demonstrated 66% and 92% reductions in *Stxbp1* and STXBP1 levels, respectively, after 72 hours of *Stxbp1* siRNA reverse-transfection (*P* < 0.05-0.01). *Stxbp1* KD did not alter the expression of other key SNARE transcripts (*Stx1a*, *Stx1b*, *Stx2-4*, *Stxbp2*, *Stxbp3*; Fig. S2 in File Inventory (29)).

**Figure 4.**
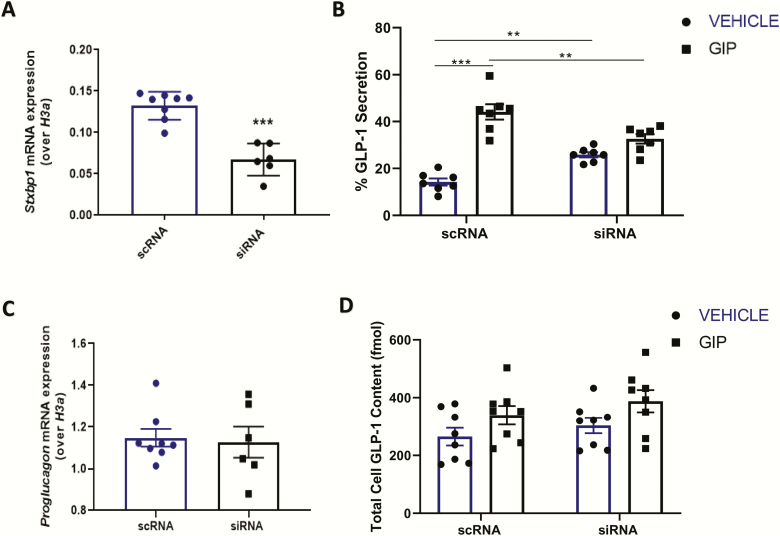
**Knockdown of *Stxbp1* in the mGLUTag L-cell impairs GLP-1 secretion in vitro.** mGLUTag cells were treated with siRNA for *Stxbp1* or scRNA (control), synchronized and then either analyzed at the 8-hour time-point by RT-quantitative PCR for (A) *Stxbp1* and (C) *Gcg*, or were treated with 10^–7^ M GIP for 2 hours followed by (B) a GLP-1 secretion assay and (D) determination of total cell content of GLP-1. (n = 7–8). **P < 0.01, ***P < 0.001.

*Stxbp1* KD in synchronized mGLUTag L-cells achieved a 49% reduction in *Stxbp1* expression at the 8-hour time-point (*P* < 0.001; Fig. 4A), at which time a 2-hour GLP-1 secretion assay was conducted. ScRNA-transfected, control cells demonstrated a 3.1-fold increase in GLP-1 secretion in response to the known GLP-1 secretagogue, glucose-dependent insulinotrophic polypeptide (GIP (6, 11, 23)), *P* < 0.001; Fig. 4B). Basal GLP-1 secretion was significantly increased following *Stxbp1* KD, by 10.9% (*P* < 0.01). In contrast, GIP-stimulated GLP-1 secretion was abolished by the KD, and the overall response was reduced by 74.9% as compared to GIP-stimulated control cells (*P* < 0.01; Fig. 4B). This impairment in secretory response was not due to altered GLP-1 levels, as both *Gcg* expression (the gene encoding GLP-1) and the total GLP-1 content of the cultures did not differ between siRNA- and scRNA-treated cells (Fig. 4C, D). Collectively, these findings demonstrate an essential role for Stxbp1 in peak GLP-1 secretion by synchronized mGLUTag L-cells.

### KO of Stxbp1 in the primary L-cell impairs GLP-1 secretion in vivo

**Figure 5.**
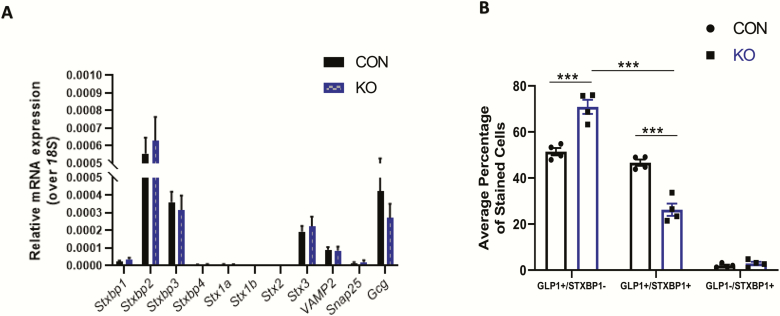
**Validation of the L-cell *Stxbp1* KO *in vivo*.** (A) Colonic mucosal scrapes from L-cell *Stxbp1* KO and control mice were analyzed by RT-qPCR for expression of various SNARE proteins as well as *Gcg* (n = 8–9). (B) Colonic sections from L-cell *Stxbp1* KO and control mice were analyzed by immunostaining for coexpression of STXBP1 and GLP-1 (n = 4). ***P < 0.001. CON, control; KO, knockout.

Inducible *Stxbp1* KO mice were developed to determine the role of Stxbp1 in GLP-1 release by the primary L-cell (Fig. S1 in File Inventory (29)). RT-qPCR of colonic mucosa did not demonstrate any change in *Stxbp1* expression in the KO mice (Fig. 5A), likely because of the rarity of L-cells in the intestine (less than 0.5% of total epithelial cells (44)). The mice also did not demonstrate changes in mucosal transcripts for *Gcg* or other SNARE isoforms (Fig. 5A), or in body weight or intestinal weights and lengths (Fig. S3 in File Inventory (29)). Nonetheless, successful reduction of STXBP1 expression in the L-cell was confirmed by immunofluorescence, with a 43.8% decrease in the proportion of GLP-1^+^/STXBP1^+^ cells in the colon of KO mice (Fig. 5B).

**Figure 6.**
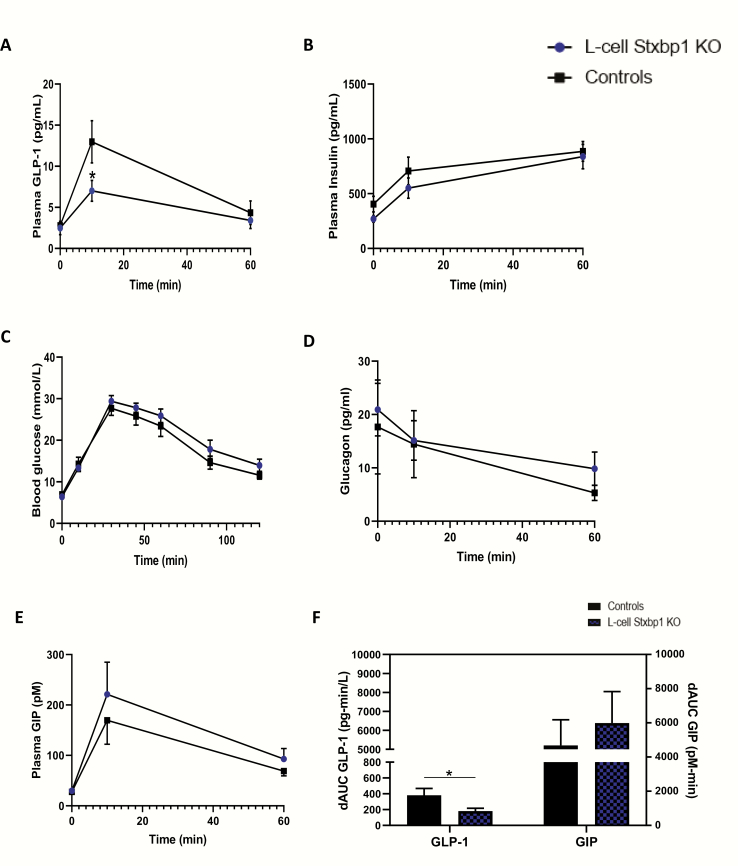
**KO of *Stxbp1* in the primary L-cell impairs GLP-1 secretion in vivo.** (A) Plasma GLP-1, (B) plasma insulin, (C) blood glucose, (D) plasma glucagon, (E) plasma GIP levels and comparative delta area-under-the-curve (dAUC) for (F) the GLP-1 and GIP responses, following a 4 g/kg OGTT administered at ZT14 (8 pm) to L-cell *Stxbp1* KO and control mice (n = 9, A-C; n = 3–5, D-E). *P < 0.05. GIP, glucose-dependent insulinotrophic polypeptide; GLP-1, glucagon-like peptide-1; KO, knockout; OGTT, oral glucose tolerance test.

In contrast to the findings following *Stxbp1* KD in the mGLUTag L-cells, 4-hour fasting plasma GLP-1 concentrations were not altered by L-cell *Stxbp1* KO, nor were any changes in fasting plasma insulin or blood glucose levels detected (Fig. 6A–C). However, whereas GLP-1 levels rose significantly at t = 10 minutes in control animals in response to the oral glucose load (by 368.9%; *P* < 0.001), the response in L-cell *Stxbp1* KO mice was markedly impaired (by 50.1%; *P* < 0.01). This loss of GLP-1 did not result in any significant dampening of insulin secretion or an impairment in glucose tolerance during the OGTT. No sex differences were observed between males and females for any of the measured parameters (data not shown).

Given that the *Gcg* promoter used to generate the *Stxbp1* KO is expressed not only in the intestinal L-cell, but also the pancreatic α-cell (45, 46) (Supplemental Methods in File Inventory (29)), plasma glucagon levels were determined in a subset of the mice. However, no differences were observed in glucagon concentrations between KO and control animals under either basal conditions or following the OGTT (Fig. 6D). Plasma GIP levels were also determined because increased secretion of this incretin hormone is known to compensate for the constitutive absence of GLP-1 signaling in mice (47, 48). Although slightly elevated at both 10 and 60 minutes, plasma GIP levels did not differ significantly between KO and control animals (Fig. 6E). Nonetheless, the delta-area under the curve for the overall GLP-1 response was decreased in the KO mice (by 54%; *P* < 0.05), and this was paralleled by a 23% increase in the delta-area under the curve for GIP (Fig 6F).

### KO of Stxbp1 in the L-cell impairs GLP-1 secretion ex vivo

**Figure 7.**
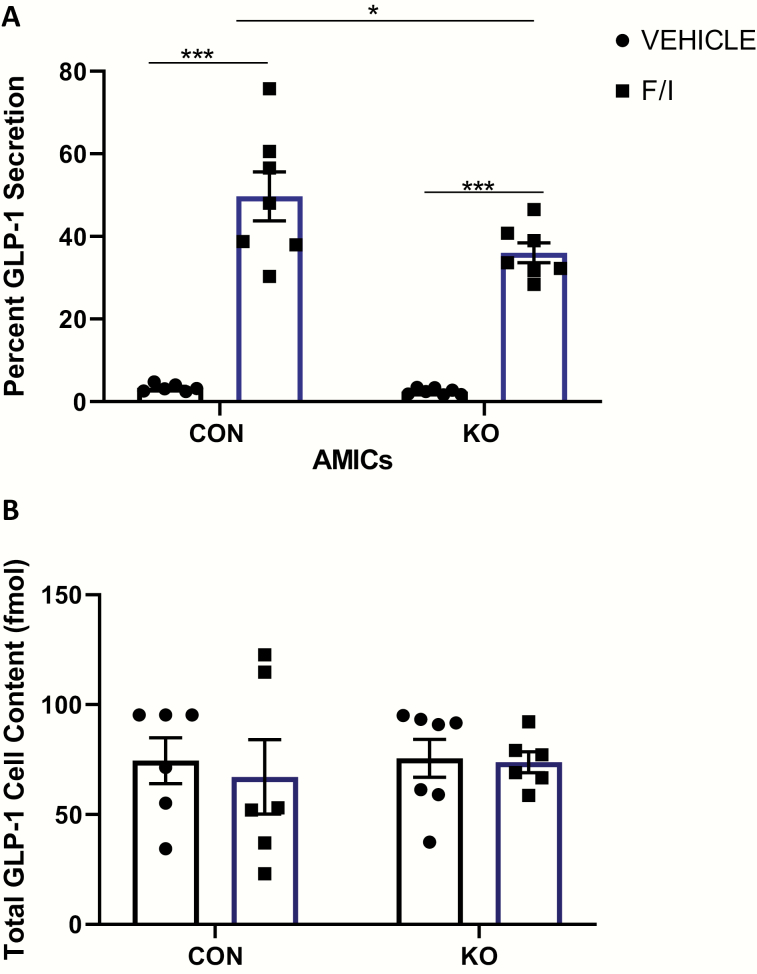
**KO of *Stxbp1* in the primary L-cell impairs GLP-1 secretion ex vivo.** GLP-1 secretion assay in primary adult mouse ileal crypt cultures from L-cell *Stxbp1* KO and control mice. Cells were treated for 2 hours with 50 µM forskolin (F) plus 50 µM IBMX (I) or vehicle alone (control). (A) Percent GLP-1 secretion and (B) total cell content of GLP-1. (n = 6–7). *P < 0.05, ***P < 0.001. CON, control; IBMX, 3-isobutyl-1-methylxanthine; KO, knockout.

Finally, primary ileal crypt cultures derived from *Stxbp1* KO mice also demonstrated no differences in basal GLP-1 secretion compared with cultures from control mice (Fig. 7). However, a 27.5% reduction in 2-hour forskolin/IBMX-stimulated GLP-1 secretion was observed in the *Stxbp1* KO cultures as compared to those derived from control animals (*P* < 0.05), despite similar GLP-1 content.

## Discussion

The push to develop new classes of GLP-1-based therapies has led to identification of novel secretagogues regulating GLP-1 release from the L-cell (2). As an alternative approach (49), the pathway governing exocytosis of the GLP-1-containing secretory granules may serve as a potential therapeutic target. Studies focused on the role of the SNARE proteins in GLP-1 secretion are currently in early stages, with only the core proteins, VAMP2 and syntaxin1a, and the accessory proteins, synaptotagmin-7 and secretagogin, being identified to date as regulators of GLP-1 secretion (10, 15–17). The results of the present study contribute to this growing body of work, by identifying the regulatory SNARE protein, Stxbp1, as a novel regulator of GLP-1 secretion. Using in vitro, in vivo, and ex vivo models, this study demonstrated that the loss of Stxbp1 in L-cells leads to a significant reduction in stimulated GLP-1 secretion. Furthermore, a relationship between the expression of Stxbp1 and the clock gene, Bmal1, identified Stxbp1, along with secretagogin (10), as a putative determinant of the circadian pattern of GLP-1 secretion.

Although L-cell Stxbp1 deficiency impaired stimulated GLP-1 secretion in all models tested, the decrease in the GLP-1 response to an OGTT, unexpectedly, did not result in any significant alterations in either plasma insulin levels or glycemia. These findings stand in contrast to the reduced insulinemia and impaired glucose tolerance observed with the loss of GLP-1 secretion in the syntaxin1a KO mouse (16). However, the syntaxin1a KO mouse was generated using the *Villin*-*CreER*^*T2/+*^ construct, which targets all intestinal epithelial cells, resulting in impaired secretion of GIP which, presumably, contributed to the phenotype. Conversely, increased GIP secretion compensates for chronic loss of GLP-1 action, accompanied by enhanced β-cell sensitivity to the insulinotrophic actions of GIP (47, 48, 50). Although we did not determine GIP receptor signaling in the present acute study, the results are consistent with an adaptive role for GIP in limiting hyperglycemia in the L-cell *Stxbp1* KO mice. Importantly, given that the expression of the *Gcg*-*CreER*^*T2/+*^ construct is not constrained to the intestinal L-cell, but also occurs in the pancreatic α-cell (45, 46), as well as the reported expression of Stxbp1 in the α-cell (20, 51, 52), use of this construct to generate the L-cell *Stxbp1* KO mice was plausibly predicted to affect the secretion of glucagon. However, although plasma glucagon levels were suppressed normally during the OGTT, no change in fasting glucagon levels was observed, suggesting that Stxbp1, which is expressed at lower levels in α compared with β and δ cells (53) plays a minor role and/or is redundant for glucagon secretion. Finally, although possible effects of *Stxbp1* KO in *Gcg*-expressing neurons of the CNS could not be accounted for in the present study, no changes in body weight were noted in the experimental animals.

Importantly, STXBP1 expression was detected in some, but not all ileal L-cells in both mice and humans, consistent with recent studies demonstrating that L-cells are not identical either along the length of the intestinal tract or across the crypt-villus axis (41, 42, 54). Indeed, our recent study has demonstrated that differential receptor expression by subpopulations of colonic L-cells results in profound differences in sensitivities to known secretagogues (41). Hence, the marked effect of *Stxbp1* KO on the L-cell response to an OGTT in vivo and to forskolin ex vivo suggest a significant role for this regulatory SNARE protein in GLP-1 secretion by a unique population of intestinal L-cells. These findings further raise the intriguing possibility that Stxbp1 may serve as a marker for secretagogue-specific L-cell responses.

STXBP1 was found to translocate within the mGLUTag L-cells upon stimulation with forskolin, toward the cytoplasm and the cell membrane. Nuclear expression of STXBP1 has been reported in both neurons and PC12 cells, although its function therein remains unknown (55). However, in many neuro/endocrine cell types, STXBP1 binds to Syntaxin1 during its translocation through the cytosol to the plasma membrane, maintaining Syntaxin1 in its “closed” conformation to prevent premature SNARE fusion. However, at the cell membrane, STXBP1 primes core SNARE fusion by inducing the “open” conformation of syntaxin1 (21, 56, 57). As syntaxin1a is expressed by intestinal L-cells (16), and STXBP1 interacts with Syntaxin1a in the mGLUTag L-cell line (15), these results are consistent with a role for Stxbp1 as a regulator of Syntaxin1a function in the L-cell.

In conclusion, the results of this study demonstrate a key role for the regulatory SNARE protein, STXBP1, in GLP-1 secretion from the intestinal L-cell. Consistent with data implicating Stxbp1 in the circadian secretion of glucagon and insulin (20), the present study identifies the *Stxbp1* promoter as a time-dependent target of BMAL1, and demonstrates the existence of a circadian rhythm in Stxbp1 transcript and protein expression in L-cells. When taken with reports that *Stxbp1* expression is reduced in islets from patients with type 2 diabetes, as well as from the Goto-Kakizaki rat model of type 2 diabetes (52, 58, 59), these findings suggest Stxbp1 as a potential target to enhance not only insulin secretion, but also GLP-1 release.

## Data Availability

All data generated or analyzed during this study are included in this published article or in the data repositories listed in References.

## Abbreviations

ChIP: chromatin-immunoprecipitation
FBS: fetal bovine serum
GIP: glucose-dependent insulinotrophic polypeptide
GLP-1: glucagon-like peptide-1
IBMX: 3-isobutyl-1-methylxanthine
KD: knockdown
OGTT: oral glucose tolerance test
RT-qPCR: RT-quantitative PCR
scRNA: RNA
siRNA: small interfering RNA
ZT: zeitgeber time

## Additional Information

***Disclosure Summary:*** The authors have nothing to disclose.
